# Department of Dermatology

**Published:** 1893-08

**Authors:** Isadore Dyer

**Affiliations:** Lecturer and Clinical Instructor Medical Department Tulane University, Etc. New Orleans, La.


### DEPARTMENT OF DERMATOLOGY.

[Dermatological Notes by Isadore Dyer, M. D., Lecturer and Clinical Instructor Medical Department Tulane University, etc. New Orleans, La.]

TREATMENT OF EPHILIDES BY THE METHOD CALLED "ÉCORCHEMENT.—In an article read before the French Dermatological Society, from the pen of Dr. M. Van Horn, of Amsterdam, this method was discussed. It follows Unna's practice, and according to the author has been used successfully in confluent freckles, uterine chloasma, rebellious acne rosacea, scars and other disfiguring lesions, on the face especially. The author calls the process the method of Ècorchement, which means that a veritable epidermic mask comes away in a single piece and represents almost the entire epidermis. When the skin of the face has been separated in this way, detached superficially, the skin underneath appears free from blemish, and freed entirely from the objectionable or disfiguring lesions.

This result is obtained in about a week at the cost of considerable pain, like that of a blister. In the case of pigmentation of chloasma, a single application suffices. It must be repeated in deeper seated and more obstinate cases; the cicatrices following small-pox, for example. Here the object is, of course, to diminish as much as possible the differences in color and elevation between the cicatrices and the healthy skin.

Here is the method:—Several times a day the patient is instructed to apply the following to the skin to be treated:

| | | | |
|---|---|---|---|
| ℞ | Resorcin........................ | 40 | grammes |
| | Oxide of zinc...... .......... | 10 | " |
| | Anhydrous silicylic acid..... ... | 2 | " |
| | Lard............................ | 20 | " |
| | Olive oil ...................... | 8 | " |

M.

In three or four days the skin becomes dry and chapped. Now is the time to apply the treatment, which consists of the following gelatinous paste:

| | | | |
|---|---|---|---|
| ℞ | White gelatine .................... | 4 | grammes |
| | Oxide of zinc .................... | 3 | " |
| | Glycerine .................... | 5 | " |
| | Distilled water .................... | 8 | " |

M.

This should be applied hot, and covered with lint to avoid sticking. In a few days the old layer of the epidermis becomes detached and together with the dried paste can be readily removed with a pair of scissors.

This treatment has been used successfully in the clinics of the St. Louis Hospital in Paris, but it is doubtful whether the pain incurred is justified by the result obtained.—*Annales de Derm. et Syphilog.*, May, 1893, p. 577.

P. H. PYE-SMITH ON DISEASES OF THE SKIN. Lea Bros. Philadelphia, 1893.—Compared with Dr. Crocker's recent and most modern edition on this branch, this little contribution suffers. Considered alone, however, there are many points worthy of criticism.

The work is practical in the treatment suggested, and in the methods of diagnosis. The suggestions for the classification of skin diseases are confusing and too elaborate to be useful. They differ, moreover, so far from the lines usually accepted and followed that so radical an innovation must be condemned. Dr. Pye-Smith's book is not modern, and is not complete, many important skin diseases having been omitted. The work is full of clinical suggestions. The diagrams of the localization of certain skin affections is worth noticing, and the article on eczema is well prepared. It can scarcely be commended as a text-book, but the specialist will be glad to read a work in which the author predominates, and which differs so much from the recent works on the same subject.

THE ELIMINATION OF MERCURY.—Linden: Untersuchungen über die Resorption und Elimination des Quecksilbers. (Ergänzungshefte zum Archiv. f. Dermatologie und Syphilis, 1892, No. 2, p. 226.)

The author arrives at the following conclusions: In a mercurial treatment the mercury is eliminated in a regular manner as long as it is present in the system. The quantity of mercury excreted depends upon the amount ingested and the time elapsed since treatment was stopped. After a single injection of the salicylate of mercury, the mercury is found very quickly in the urine, several hours after, even. There is more eliminated durring the first and second day. After that it diminishes rapidly, and ordinarily one cannot find it at the beginning of the second week. After a treatment with injections of a large amount of salicylate of mercury in a relatively short time, one can confirm the presence of the mercury in the urine after a lapse of time notably longer than after a single injection. The proportion of mercury remains large up to the end of the first week, when it begins to diminish. It can also often be missed after as small an injection as five per cent. During subsequent weeks the diminution continues, until one can find in the fourth week the mercury in the proportion of seventy per cent. In the second month it is not found in larger proportion than twenty-four per cent. for all the analyses. After a treatment with inunctions, the mercury does not appear much later in the urine, that is to say, after one or two weeks of treatment. Later, it is found all through the treatment and the presence is determined four weeks or so after treatment is stopped. After a course of internal treatment the mercury appears in the urine much later than after the injection treatment, not until about the fifth or sixth day, and proportionately more feeble. The author has failed to discover the mercury nineteen days after the treatment was finished.

The most of the mercurial preparations employed provoke in the tissues a marked irritation due to the coagulation of the albumen. They cause infiltration and often abscesses. Liebrich insists upon the necessity of choosing for injection only such mercurials as do not form combinations in the tissues, or only those which are readily soluble. The salicylate seems to be more soluble than the other salts and it remains dissolved in sodium chloride solution. This solution is found in the system. The salicylate, therefore, must be absorbed. Clinical experience has confirmed the justice of this opiniou, that the mercurial salt is absolved as promptly as other soluble preparations, without causing marked irritation. The rapidity of the absorption is evidenced by the salivation which appears often one hour after the injection, and by the prompt modification of the system often seen on the second day.

Different opinions obtained as to the advantage of a more or less rapid elimination of mercury. Nevertheless, a prompt absorption and subsequent rapid elimination would seem to account for the fact that the author had never observed mercurialism, in spite of prolonged treatment by injection with doses of ten centigrammes. To obtain an action as rapid and as durable as possible without provoking hydrargyrism, the author has found that it was preferable to employ the salicylate of mercury in the dosage of ten centigrammes and to make the three or four first injections at intervals of four or five days. Subsequent injections were made seven to nine days apart.—*M. Doyon in "Annales" for May*, p. 681.

